# Consensus Tracking of Nonlinear Agents Using Distributed Nonlinear Dynamic Inversion with Switching Leader-Follower Connection

**DOI:** 10.3390/s22239537

**Published:** 2022-12-06

**Authors:** Sabyasachi Mondal, Antonios Tsourdos

**Affiliations:** School of Aerospace, Transport and Manufacturing (SATM), Cranfield University, Cranfield MK43 0AL, UK

**Keywords:** consensus tracking, distributed nonlinear dynamic inversion, leader–follower consensus

## Abstract

In this paper, a consensus tracking protocol for nonlinear agents is presented, which is based on the Nonlinear Dynamic Inversion (NDI) technique. Implementation of such a technique is new in the context of the consensus tracking problem. The tracking capability of nonlinear dynamic inversion (NDI) is exploited for a leader-follower multi-agent scenario. We have provided all the mathematical details to establish its theoretical foundation. Additionally, a convergence study is provided to show the efficiency of the proposed controller. The performance of the proposed controller is evaluated in the presence of both (a) random switching topology among the agents and (b) random switching of leader–follower connections, which is realistic and not reported in the literature. The follower agents track various trajectories generated by a dynamic leader, which describes the tracking capability of the proposed controller. The results obtained from the simulation study show how efficiently this controller can handle the switching topology and switching leader-follower connections.

## 1. Introduction

Multiple UAV or multi-agent operation has been an exciting research area for years. Multi-agent systems (MASs) play an important role in executing complex tasks, which are usually difficult for a single UAV or agent. Examples of MASs applications are cooperative mobile robotics [1], sensory networks [2], flocking [3], formation control of robot teams [4], rendezvous of multiple spacecraft [5], etc. Agents share information over a communication network and take appropriate control action to agree on a decision, i.e., they achieve consensus. The control action is generated by consensus protocols designed using control theory. Researchers have proposed a variety of consensus protocols to solve different categories of consensus problems considering linear and nonlinear agents, like communication issues (switching topology [6,7,8], delays [9,10,11], noise [12,13,14]), disturbance [15,16], and fault [17,18].

A significant number of these protocols achieve the consensus with a single value, which primarily depends on the initial values of the agents. However, in a real-world scenario, the agents may need to converge to a time-varying consensus value, which is available to a few agents of networked MASs. This problem can be categorized as a consensus tracking problem (also known as a leader–follower consensus problem because a leader agent provides the time-varying values). Leader–follower consensus protocols have been proposed to solve this problem. In [19], the authors considered the nearest neighborhood principle and showed that all agents’ states converged to the leader’s state if the agents were jointly connected to the leader. However, this scheme was too restrictive. Ren and Beard [20] addressed the same problem in [19] with directed topology and relaxed restriction on the topology. Ren [21] showed that the consensus protocol of a proportional and derivative type could track a time-varying reference state of a virtual leader, but a proportional-like consensus protocol cannot do it. Peng et al. [22] solved a leader–following consensus problem. The leader agent has varying velocity and time-varying delays. Cao et al. [23] presented leader–follower consensus using a variable structure method. Hong et al. [24] proposed a distributed output regulation algorithm for linear agents. In [25], the authors solved a consensus problem for unknown systems. Wang et al. [26] used a distributed observer to solve an adaptive leader–follower consensus problem for higher-order agents. In [27], the authors addressed fixed-time event/self-triggered leader–follower consensus problems for networked multi-agent systems having nonlinear dynamics. In [28], the authors proposed distributed adaptive protocol for cooperative tracking problem considering pure relative output information. Guo et al. [29] discussed a fixed-time consensus tracking problem of nonlinear agents via discontinuous protocols. In [30], the adaptive consensus tracking control problem of nonlinear multi-agent systems (MASs) is solved using a robust adaptive event-triggered sliding-mode control method. Additionally, the authors considered unknown perturbations and limited network bandwidth in the problem.

One of the significant events that cause the tracking failure is the actuator fault. There exist a few papers that addressed the actuator fault in the consensus tracking problem. Qin et al. [31] implemented sliding mode control to solve the consensus tracking problem of nonlinear agents with actuator faults. They also considered disturbance in their study. Mu et al. [32] proposed an event-triggered control strategy to solve the leader–following consensus problem of agents with time-varying actuator faults. Xia et al. [33] presented a fault-tolerant fuzzy tracking controller for nonlinear agents subject to actuator failures and external disturbances. Gong et al. [34] studied an adaptive cooperative fault-tolerant supervisory control problem for nonlinear leader–follower agents with unknown control coefficients and actuator faults. More results can be found in [35,36]. Along with the actuator fault, switching topology is another event that is practical and causes difficulties during the consensus process. A few works have been reported in the literature where the effect of switching topology in tracking is studied. Wen et al. [6] presented consensus Tracking of agents having Lipschitz node dynamics and switching Topologies. Wang et al. [37] addressed a $H_{\infty }$ consensus tracking control problem for linear agents. They considered switching topology and disturbances in their study. Razaq et al. [38] presented a leader-based consensus of one-sided Lipschitz (OSL) agents under switching graphs and input saturation. It can be mentioned that there exist a small number of papers that discussed consensus tracking considering both the switching topology and actuator fault. Sader et al. [39] presented the consensus tracking problem of agents’ nonlinear function, exogenous disturbances, and actuator faults. They considered the switching communication topologies in their study. Liu et al. [40] designed a distributed fault-tolerant consensus tracking control for multi-agent systems with actuator faults considering both fixed and switching topologies. Additionally, Cao et al. [41] solved the same problem of consensus tracking control of stochastic agents with actuator fault under randomly switched topology.

It can be mentioned that, in the leader–follower or consensus tracking problem, the followers are connected to a few agents of the network. The connection between these followers and the leader can also change in a similar way in which the switching topology occurs. However, this leader–follower switching connection is not addressed in any paper. We will address this problem along with the actuator fault in this paper.

All of these papers implemented linear and nonlinear control theory to design the controller. There exists a control technique that is very efficient in designing controllers for nonlinear plants. The philosophy behind NDI is to use feedback linearization theory to remove the nonlinearities in the plant. Additionally, the response of the closed-loop plant is similar to a stable linear system. There are many advantages to using an NDI controller, e.g., (a) closed-form control expression, (b) easy mechanization, (c) global exponential stability, (d) inclusion of nonlinear kinematics in plant inversion, and (e) minimization of the need for individual gain tuning or gain scheduling. It has been used to design controllers in various applications. In [42], the authors designed an NDI-based flight controller. In [43], NDI controller was implemented for autonomous landing of UAV. Lifeng et al. [44] used Improved Dynamic Inversion to design trajectory tracking control for a quadrotor. In [45], the authors used an NDI controller to present a flying formation scheme. The follower UAVs are used to track the desired attitude commanded by the leader. The attitude control of a flexible aircraft was described using dynamic inversion by Caverly et al. [46]. Another example of using NDI to solve an attitude control problem of a hovering quad tiltrotor eVTOL Vehicle was presented by Lombaerts et al. [47]. In [48], the authors used NDI to track the angular reference rates obtained from the guidance command in a missile guidance problem. In [49], the authors presented a Nonlinear Dynamic Inversion (NDI) based flight controller for a VTOL aircraft, including transition maneuvers. They used virtual controls, generalized forces and moments to control its longitudinal motion. A fault-tolerant control (FTC) scheme was proposed by Ma et al. [50]. The scheme was based on extended state observer (ESO) and nonlinear dynamic inversion (NDI). In [51], trajectory generation and control architecture for a fully autonomous autorotative flare are proposed. These flare trajectories are tracked by a nonlinear dynamic inversion (NDI) control law.

These papers present the implementation of dynamic inversion to design a controller for a single platform. Mondal et al. [14] proposed a distributed consensus protocol based on NDI and named it Distributed NDI or DNDI. It has been implemented to solve consensus problems with actuator fault [18], external disturbances [16], and bipartite consensus [52]. In this paper, we have proposed a variety of DNDI that exploits the tracking capability of NDI and successfully solves a leader–following consensus tracking problem, which is different from leaderless consensus in terms of concept and formulation. We have evaluated the performance of the proposed controller in the presence of both (a) switching topology among the agents and (b) switching connections between the leader and the followers.

The contribution in this paper is given as follows.

- Distributed Nonlinear Dynamic Inversion (DNDI) based control protocol is designed to address the consensus tracking problem of nonlinear agents for the first time. This is novel because we exploited the tracking capability of nonlinear dynamic inversion (NDI) for a leader-follower multi-agent scenario.
- Detailed mathematical derivation of the controller is provided.
- Mathematical details for convergence study are presented, which gives proof of its correctness.
- We have considered the presence of both (a) switching topology among the agents and (b) switching connection between the leader and the followers to make the scenario more realistic. This is new in the context of the consensus tracking problem.
- Realistic simulation study shows the accuracy of the proposed controller. Different types of leader trajectories are generated to demonstrate the tracking capability of the proposed controller.

The rest of the paper is organized as follows. In Section 2, the preliminaries are given. In Section 3, the problem description is presented. Mathematical details of tracking DNDI for leader–follower consensus tracking is shown in Section 4. The convergence study of tracking DNDI is presented in Section 5. Simulation results are shown in Section 6, and Section 7 gives the conclusion.

## 2. Preliminaries

In this section, we have presented a few topics that are relevant to this study.

### 2.1. Consensus Tracking of Multiple Agents

Let us consider *N* nonlinear agents connected by a communication topology. The agents (called followers) need to track the trajectory of a leader $X_{L}\left(t\right)$, which is connected to a few agents of the networked agents. If the followers’ states, i.e., $X_{i}\left(t\right);\;i=1,2,\ldots ,N$ achieve the consensus and track the leader’s states, i.e., if for any initial conditions $\lim_{t\to \infty }\left|X_{i}\left(t\right)\to X_{L}\left(t\right)\right|=0$, the followers are considered to achieve consensus tracking.

### 2.2. Graph Theory

The communication among the agents can be represented by a weighted graph written by G={V,E}. The vertices $V=\{v_{1},v_{2},\ldots ,v_{N}\}$ of the graph are used to represent the agents. The set of edges, i.e., E⊆V×V, shows the communication among the agents. The elements of weighted adjacency matrix $A=\left[a_{ij}\right]\in {ℜ}^{N\times N}$ of G are given by $a_{ij}>0$ if $(v_{j},v_{i})\in E$, otherwise $a_{ij}=0$. There is no self-loop in the graph, i.e., the diagonal elements of the adjacency matrix A as zero ($a_{ii}=0$). The degree matrix is represented as $D\in {ℜ}^{N\times N}=diag\left\{d_{1}\;d_{2}\;\ldots d_{N}\right\}$, where $d_{i}=\sum_{j\in N_{i}}a_{ij}$. The Laplacian matrix is written as L=D−A. In this paper, we consider the topology G of the network as undirected (i.e., $a_{ij}=a_{ji}$) and connected ($v_{i},v_{j}\in V$, there exists a path from $v_{i}$ to $v_{j}$).

### 2.3. Switching Leader-Follower Connection

In this paper, we have evaluated the performance of the proposed controller in the presence of a switching leader–follower connection along with the switching topology among the followers. In case of a consensus tracking problem, the leader’s state information is available to a few follower agents. Let us consider the leader’s state information is available to *p* agents, p⊂N, where *N* is the total number of followers. At any time $t\geq t_{0}$, the recipient followers are changed (some of them or all), and the leader’s information is available to *q* agents (p=q or p≠q).

### 2.4. Lemma

The useful lemmas used in this paper are given as follows.

**Lemma** **1** ([20]). *The Laplacian matrix L in an undirected graph is semi-positive definite, it has a simple zero eigenvalue, and all the other eigenvalues are positive if and only if the graph is connected. Therefore, L is symmetric and it has N non-negative, real-valued eigenvalues $0=\lambda_{1}\leq \lambda_{2}\leq \ldots \leq \lambda_{N}$.*

**Lemma** **2** ([53]). *Let $\psi_{1}\left(t\right),\psi_{2}\left(t\right)\in R^{m}$ be continuous positive vector functions, by Cauchy inequality and Young’s inequality, there exists the following inequality:*

(1) $$\begin{array}{ccc} \psi_{1}\left(t\right)\psi_{2}\left(t\right) & \leq & \|\psi_{1}\left(t\right)\|\|\psi_{2}\left(t\right)\|\\ & \leq & \frac{\|\psi_{1}{\left(t\right)\|}^{\bar{\lambda }}}{\bar{\lambda }}+\frac{\|\psi_{2}{\left(t\right)\|}^{\bar{\zeta }}}{\bar{\zeta }} \end{array}$$

*where*

$$\frac{1}{\bar{\lambda }}+\frac{1}{\bar{\zeta }}=1$$

**Lemma** **3** ([54]). *Let R(t)∈ℜ be a continuous positive function with bounded initial R(0). If the inequality holds $\dot{R}\left(t\right)\leq -\nu R\left(t\right)+\varsigma$ where, ν>0,ς>0, then the following inequality holds.*

(2) $$R\left(t\right)\leq R\left(0\right)e^{-\nu t}+\frac{\varsigma }{\nu }\left(1-e^{-\nu t}\right)$$

## 3. Problem Formulation

In this section, the problem definition is given. The objective is to design a consensus tracking protocol that enables a class of nonlinear agents’ (follower) states $X_{i}\left(t\right);\;i=1,2,\ldots ,N$ to achieve the consensus and track the desired signal ($X_{L}\left(t\right)$) generated by a leader agent, i.e., $X_{i}\left(t\right)\to X_{L}\left(t\right)$. The *i*th follower agent is described by

(3) $$\begin{array}{ccc} {\dot{X}}_{i} & = & f\left(X_{i}\right)+g\left(X_{i}\right)U_{i} \end{array}$$

(4) $$\begin{array}{ccc} Y_{i} & = & X_{i} \end{array}$$

where, $X_{i}\in {ℜ}^{n}$, $U_{i}\in {ℜ}^{n}$ are states and control, respectively. *f* is a continuously differentiable vector-valued function representing the nonlinear dynamics.

**Assumption** **A1.** *The matrix $g(X_{i})$ is invertible for all time.*

*The leader dynamics is given by*

(5) $${\dot{X}}_{L}\left(t\right)=f_{L}\left(X_{L}\left(t\right),t\right)$$

*where,*$X_{L}\in {ℜ}^{n}$. $f_{L}$*is piecewise continuous in t.*

**Assumption** **A2.** *$X_{L}\left(t\right)$ and ${\dot{X}}_{L}\left(t\right)$ are assumed to be bounded.*

It can be mentioned that the leader’s state information is available to a few agents of the networked agents.

## 4. Distributed Nonlinear Dynamic Inversion for Consensus Tracking

Considering the agent (Equations (3) and (4)) and leader dynamics (Equation (5)), the consensus tracking error of $i^{th}$ agent (scalar n=1) is given by

(6) $$e_{i}=\sum_{j\in N_{i}}a_{ij}\left(x_{i}-x_{j}\right)+\beta_{i}\left(x_{i}-x_{L}\right)$$

Simplifying Equation (6), we obtain

(7) $$e_{i}=\left(d_{i}+\beta_{i}\right)x_{i}-a_{i}X-\beta_{i}x_{L}$$

where $X\in {ℜ}^{N}$, $x_{L}$ defines the state of a scalar agent, and $\beta_{i}$ shows if *i*th agent is connected to the leader. The tracking error is given for the agents with state vector $X_{i}\in {ℜ}^{n};n>1$.

(8) $$E_{i}=\left({\bar{d}}_{i}+{\bar{\beta }}_{i}\right)X_{i}-{\bar{a}}_{i}X-{\bar{\beta }}_{i}X_{L}$$

where $E_{i}\in {ℜ}^{n}$, ${\bar{d}}_{i}=\left(d_{i}⊗I_{n}\right)\in {ℜ}^{n\times n}$, ${\bar{a}}_{i}=\left(a_{i}⊗I_{n}\right)\in {ℜ}^{n\times nN}$, ${\bar{\beta }}_{i}=\left(\beta_{i}⊗I_{n}\right)\in {ℜ}^{n\times n}$, $X_{L}\in {ℜ}^{n}$, and $X={\left[X_{1}^{T}\;X_{2}^{T}\;\ldots \;X_{N}^{T}\right]}^{T}\in {ℜ}^{nN}$. We enforce the first-order error dynamics as follows.

(9) $${\dot{E}}_{i}+K_{i}E_{i}=0$$

Differentiation of Equation (9) gives

(10) $$\begin{array}{ccc} {\dot{E}}_{i} & = & \left({\bar{d}}_{i}+{\bar{\beta }}_{i}\right){\dot{X}}_{i}-{\bar{a}}_{i}\dot{X}-{\bar{\beta }}_{i}{\dot{X}}_{L}\\ & = & \left({\bar{d}}_{i}+{\bar{\beta }}_{i}\right)\left[f\left(X_{i}\right)+g\left(X_{i}\right)U_{i}\right]-{\bar{a}}_{i}\dot{X}-{\bar{\beta }}_{i}{\dot{X}}_{L} \end{array}$$

The expressions of $E_{i}$ and ${\dot{E}}_{i}$ are substituted in Equation (9) to obtain

(11) $$\left({\bar{d}}_{i}+{\bar{\beta }}_{i}\right)\left[f\left(X_{i}\right)+g\left(X_{i}\right)U_{i}\right]-\bar{a}\dot{X}-{\bar{\beta }}_{i}{\dot{X}}_{L}+K_{i}\left(\left({\bar{d}}_{i}+{\bar{\beta }}_{i}\right)X_{i}-{\bar{a}}_{i}X-{\bar{\beta }}_{i}X_{L}\right)=0$$

Control $U_{i}$ of *i*th agent is obtained by simplifying Equation (11) as follows.

(12) $$U_{i}={\left(g\left(X_{i}\right)\right)}^{-1}[-f\left(X_{i}\right)+{\left({\bar{d}}_{i}+{\bar{\beta }}_{i}\right)}^{-1}({\bar{a}}_{i}\dot{X}+{\bar{\beta }}_{i}{\dot{X}}_{L}-K_{i}(\left({\bar{d}}_{i}+{\bar{\beta }}_{i}\right)X_{i}-{\bar{a}}_{i}X-{\bar{\beta }}_{i}X_{L}))]$$

## 5. Convergence Study of DNDI for Consensus Tracking

The convergence study of DNDI is presented here. We define a smooth scalar function:

(13) $$\tilde{V}=\frac{1}{2}X^{T}\left(\tilde{L}⊗I_{n}\right)X$$

$\tilde{L}⊗I_{n}$ can be represented by

(14) $$\tilde{L}⊗I_{n}=\tilde{S}\Omega {\tilde{S}}^{T}$$

where, $\tilde{S}\in {ℜ}^{nN\times nN}$ is the left eigenvalue matrix of $\tilde{L}⊗I_{n}$, $\Omega =\left(diag\{0,\lambda_{2}\left(\tilde{L}\right),\lambda_{3}(\tilde{L}\right),$…,$\lambda_{N}\left(\tilde{L})\}⊗I_{n}\right)\in {ℜ}^{nN\times nN}$ is eigenvalue matrix, ${\tilde{S}}^{T}\tilde{S}=\tilde{S}{\tilde{S}}^{T}=I_{nN\times nN}$.

(15) $$\begin{array}{ccc} \tilde{V} & = & \frac{1}{2}X^{T}\left(\tilde{L}⊗I_{n}\right)X\\ & = & \frac{1}{2}X^{T}\tilde{S}\Omega {\tilde{S}}^{T}X\\ & = & \frac{1}{2}X^{T}\tilde{S}\sqrt{\Omega }\sqrt{\Omega }{\tilde{S}}^{T}X\\ & = & \frac{1}{2}X^{T}\tilde{S}\sqrt{\Omega \bar{\Omega }}\sqrt{{\bar{\Omega }}^{-1}}\sqrt{{\bar{\Omega }}^{-1}}\sqrt{\bar{\Omega }\Omega }{\tilde{S}}^{T}X\\ & = & \frac{1}{2}X^{T}\tilde{S}\Omega {\bar{\Omega }}^{-1}\Omega {\tilde{S}}^{T}X\\ & = & \frac{1}{2}X^{T}\tilde{S}\Omega \left({\tilde{S}}^{T}\tilde{S}\right){\bar{\Omega }}^{-1}\left({\tilde{S}}^{T}\tilde{S}\right)\Omega {\tilde{S}}^{T}X\\ & = & \frac{1}{2}X^{T}\left(\tilde{S}\Omega {\tilde{S}}^{T}\right)\left(\tilde{S}{\bar{\Omega }}^{-1}{\tilde{S}}^{T}\right)\left(\tilde{S}\Omega {\tilde{S}}^{T}\right)X\\ & = & \frac{1}{2}X^{T}\left(\tilde{L}⊗I_{n}\right)\Phi \left(\tilde{L}⊗I_{n}\right)X\\ & = & \frac{1}{2}E^{T}\Phi E \end{array}$$

where $\bar{\Omega }=\left(diag\left\{\lambda_{2}\left(L\right),\lambda_{2}\left(L\right),\lambda_{3}\left(L\right),\ldots ,\lambda_{N}\left(L\right)\right\}⊗I_{n}\right)\in {ℜ}^{nN\times nN}$, $E={\left[E_{1}^{T}\;E_{2}^{T}\;\ldots \;E_{N}^{T}\right]}^{T}\in {ℜ}^{nN}$, and $\Phi =\tilde{S}{\bar{\Omega }}^{-1}{\tilde{S}}^{T}\in {ℜ}^{nN\times nN}$.

**Remark** **1.** *Using Equations (13) and (15), we can write*

(16) $$\frac{\lambda_{min}\left(\Phi \right)}{2}{\left\|E\right\|}^{2}\leq V\leq \frac{\lambda_{max}\left(\Phi \right)}{2}{\left\|E\right\|}^{2}$$

(17) $$\tilde{V}=\frac{1}{2}X^{T}\left(\tilde{L}⊗I_{n}\right)X=\frac{1}{2}X^{T}E$$

**Remark** **2.** *According to Lemma 1, $\lambda_{2}>0$. Hence, $\bar{\Omega }$ is invertible.*

**Remark** **3.** *$\Phi =\tilde{S}{\bar{\Phi }}^{-1}{\tilde{S}}^{T}$ is positive definite matrix. Hence, $\tilde{V}$ is positive definite subject to consensus error and qualify for a Lyapunov function.*

Differentiating Equation (13), we get

(18) $$\dot{\tilde{V}}=X^{T}\left(\tilde{L}⊗I_{n}\right)\dot{X}=E^{T}\dot{X}=\sum_{i=1}^{N}E_{i}^{T}\left[f\left(X_{i}\right)+g\left(X_{i}\right)U_{i}\right]$$

where, $E={\left[E_{1}^{T}\;E_{2}^{T}\;\ldots \;E_{N}^{T}\right]}^{T}\in {ℜ}^{nN}$. Substituting the control $U_{i}$ expression in Equation (18) yields

(19) $$\begin{array}{ccc} \dot{\tilde{V}} & = & \sum_{i=1}^{N}E_{i}^{T}\left[{\left({\bar{d}}_{i}+{\bar{\beta }}_{i}\right)}^{-1}\left({\bar{a}}_{i}\dot{X}+{\bar{\beta }}_{i}{\dot{X}}_{L}-K_{i}E_{i}\right)\right]\\ & = & -\sum_{i=1}^{N}E_{i}^{T}{\left({\bar{d}}_{i}+{\bar{\beta }}_{i}\right)}^{-1}K_{i}E_{i}+\sum_{i=1}^{N}E_{i}^{T}{\left({\bar{d}}_{i}+{\bar{\beta }}_{i}\right)}^{-1}{\bar{a}}_{i}\dot{X}\\ & + & \sum_{i=1}^{N}E_{i}^{T}{\left({\bar{d}}_{i}+{\bar{\beta }}_{i}\right)}^{-1}{\bar{\beta }}_{i}{\dot{X}}_{L} \end{array}$$

According to Lemma 2, we can write $E_{i}^{T}{\left({\bar{d}}_{i}+{\bar{\beta }}_{i}\right)}^{-1}{\bar{a}}_{i}\dot{X}\leq \|E_{i}\left\|\;\right\|{\left({\bar{d}}_{i}+{\bar{\beta }}_{i}\right)}^{-1}{\bar{a}}_{i}\dot{X}\|$

(20) $$\leq \frac{\|E_{i}{\|}^{2}}{2}+\frac{\|{\left({\bar{d}}_{i}+{\bar{\beta }}_{i}\right)}^{-1}{\bar{a}}_{i}\dot{X}{\|}^{2}}{2}$$

and

(21) $$E_{i}^{T}{\left({\bar{d}}_{i}+{\bar{\beta }}_{i}\right)}^{-1}{\bar{\beta }}_{i}{\dot{X}}_{L}\leq \|E_{i}\left\|\;\right\|{\left({\bar{d}}_{i}+{\bar{\beta }}_{i}\right)}^{-1}{\bar{\beta }}_{i}{\dot{X}}_{L}\|\leq \frac{\|E_{i}{\|}^{2}}{2}+\frac{\|{\left({\bar{d}}_{i}+{\bar{\beta }}_{i}\right)}^{-1}{\bar{\beta }}_{i}{\dot{X}}_{L}{\|}^{2}}{2}$$

Substituting the inequality relation in Equation (19)

(22) $$\dot{\tilde{V}}\leq \sum_{i=1}^{N}\left[-E_{i}^{T}{\left({\bar{d}}_{i}+{\bar{\beta }}_{i}\right)}^{-1}K_{i}E_{i}+{\left\|E_{i}\right\|}^{2}+\frac{\|{\left({\bar{d}}_{i}+{\bar{\beta }}_{i}\right)}^{-1}{\bar{a}}_{i}\dot{X}{\|}^{2}}{2}+\frac{\|{\left({\bar{d}}_{i}+{\bar{\beta }}_{i}\right)}^{-1}{\bar{\beta }}_{i}{\dot{X}}_{L}{\|}^{2}}{2}\right]$$

Let us design the gain $K_{i}$ as follows.

(23) $$K_{i}=\left({\bar{d}}_{i}+{\bar{\beta }}_{i}\right)\left(1+\frac{\alpha_{i}}{2}\lambda_{max}\left(\Phi \right)\right)$$

Equation (22) is written as

(24) $$\begin{array}{ccc} \dot{\tilde{V}} & \leq & \sum_{i=1}^{N}[-\frac{\alpha_{i}}{2}\lambda_{max}\left(\Phi \right){\left\|E_{i}\right\|}^{2}+\frac{\|{\left({\bar{d}}_{i}+{\bar{\beta }}_{i}\right)}^{-1}{\bar{a}}_{i}\dot{X}{\|}^{2}}{2}\\ & + & \frac{\|{\left({\bar{d}}_{i}+{\bar{\beta }}_{i}\right)}^{-1}{\bar{\beta }}_{i}{\dot{X}}_{L}{\|}^{2}}{2}]\\ & \leq & -\alpha_{i}\tilde{V}+\tilde{\eta } \end{array}$$

where, $\tilde{\eta }=\frac{\|{\left({\bar{d}}_{i}+{\bar{\beta }}_{i}\right)}^{-1}{\bar{a}}_{i}\dot{X}{\|}^{2}}{2}+\frac{\|{\left({\bar{d}}_{i}+{\bar{\beta }}_{i}\right)}^{-1}{\bar{\beta }}_{i}{\dot{X}}_{L}{\|}^{2}}{2}$. Applying Lemma 3 we obtain

(25) $$\tilde{V}\leq \frac{\tilde{\eta }}{\alpha_{i}}+\left(\tilde{V}\left(0\right)-\frac{\tilde{\eta }}{\alpha_{i}}\right)e^{-\alpha_{i}t}$$

Therefore, it is clear that $\tilde{V}$ is bounded as t→∞. Moreover, we present the Uniformly Ultimate Boundedness (UUB) as follows.

Using Equations (16) and (25), and Lemma 1.2 presented by Ge et al. [54], we can write

(26) $$\frac{\lambda_{min}\left(\Phi \right)}{2}{\left\|E\right\|}^{2}\leq \tilde{V}\leq \frac{\tilde{\eta }}{\alpha_{i}}+\left(\tilde{V}\left(0\right)-\frac{\tilde{\eta }}{\alpha_{i}}\right)e^{-\alpha_{i}t}$$

We can write Equation (26) as follows.

(27) $$\begin{array}{ccc} \frac{\lambda_{min}\left(\Phi \right)}{2}{\left\|E\right\|}^{2} & \leq & \frac{\tilde{\eta }}{\alpha_{i}}+\left(\tilde{V}\left(0\right)-\frac{\tilde{\eta }}{\alpha_{i}}\right)e^{-\alpha_{i}t}\\ \left\|E\right\| & \leq & \sqrt{\frac{2\frac{\tilde{\eta }}{\alpha_{i}}+2\left(\tilde{V}\left(0\right)-\frac{\tilde{\eta }}{\alpha_{i}}\right)e^{-\alpha_{i}t}}{\lambda_{min}\left(\Phi \right)}} \end{array}$$

It can be observed that, if $\tilde{V}\left(0\right)=\frac{\tilde{\eta }}{\alpha_{i}}$, then

(28) $$\left\|E\right\|\leq \kappa^{*}$$

∀t≥0 and $\kappa^{*}=\sqrt{\frac{2\tilde{\eta }}{\alpha_{i}\lambda_{min}\left(\Phi \right)}}$. If $\tilde{V}\left(0\right)\neq \frac{\tilde{\eta }}{\alpha_{i}}$ then for any given $\kappa >\kappa^{*}$ there exist a time $\tilde{\tau }>0$ such that $\forall t>\tilde{\tau }$, ‖E‖≤κ.

(29) $$\kappa =\sqrt{\frac{2\frac{\tilde{\eta }}{\alpha_{i}}+2\left(\tilde{V}\left(0\right)-\frac{\tilde{\eta }}{\alpha_{i}}\right)e^{-\alpha_{i}T}}{\lambda_{min}\left(\Phi \right)}}$$

Therefore, we can write

(30) $$\lim_{t\to \infty }\left\|E\right\|=\kappa^{*}$$

Hence, it is proved that the error is bounded and the consensus tracking is successful.

## 6. Simulation Study

We have presented the simulation results and discussion in this section.

### 6.1. Agent Dynamics

We have considered ten agents (N=10) for simulation. Highly nonlinear terms like sin and cos are included in the agents dynamics. The dynamics for *i*th agent [14] is given in Equations (31) and (32).

(31) $$\begin{array}{ccc} {\dot{X}}_{i_{1}} & = & X_{i_{2}}\sin\left(2X_{i_{1}}\right)+U_{i_{1}} \end{array}$$

(32) $$\begin{array}{ccc} {\dot{X}}_{i_{2}} & = & X_{i_{1}}\cos\left(3X_{i_{2}}\right)+U_{i_{2}} \end{array}$$

where, $X_{i}={\left[X_{i_{1}}\;X_{i_{2}}\right]}^{T}$. The dynamics of Equations (31) and (32) are written in the form given in Equations (3) and (4) as follows.

(33) $$f\left(X_{i}\right)=\left[\begin{array}{c} X_{i_{2}}\sin\left(2X_{i_{1}}\right)\\ X_{i_{1}}\cos\left(3X_{i_{2}}\right) \end{array}\right]$$

and

(34) $$g\left(X_{i}\right)=\left[\begin{array}{cc} 1 & 0\\ 0 & 1 \end{array}\right]$$

and

(35) $$U_{i}=\left[\begin{array}{c} U_{i_{1}}\\ U_{i_{2}} \end{array}\right]$$

where $X_{i}\in {ℜ}^{2}$. The states $X_{1_{i}}$ of all the agents are denoted by $X_{1}=\left[X_{1_{1}}\;X_{2_{1}}\;\ldots \;X_{10_{1}}\right]$. Similarly, we denote $X_{2}=\left[X_{1_{2}}\;X_{2_{2}}\;\ldots \;X_{10_{2}}\right]$, $U_{1}=\left[U_{1_{1}}\;U_{2_{1}}\;\ldots \;U_{10_{1}}\right]$, and $U_{2}=\left[U_{1_{2}}\;U_{2_{2}}\;\ldots \;U_{10_{2}}\right]$. The errors in $X_{1}$ and $X_{2}$ is given by $E\;in\;X_{1}$ and $E\;in\;X_{2}$, respectively.

### 6.2. Communication Topology

The communication topology used in this simulation study is given as follows.

(36) $$A=\left[\begin{array}{cccccccccc} 0 & 0 & 1 & 1 & 1 & 1 & 0 & 1 & 1 & 1\\ 0 & 0 & 0 & 0 & 0 & 0 & 0 & 1 & 0 & 1\\ 1 & 0 & 0 & 0 & 0 & 0 & 1 & 0 & 0 & 1\\ 1 & 0 & 0 & 0 & 1 & 1 & 0 & 0 & 0 & 1\\ 1 & 0 & 0 & 1 & 0 & 1 & 0 & 1 & 0 & 0\\ 1 & 0 & 0 & 1 & 1 & 0 & 1 & 1 & 1 & 0\\ 0 & 0 & 1 & 0 & 0 & 1 & 0 & 1 & 1 & 0\\ 1 & 1 & 0 & 0 & 1 & 1 & 1 & 0 & 0 & 1\\ 1 & 0 & 0 & 0 & 0 & 1 & 1 & 0 & 0 & 1\\ 1 & 1 & 1 & 1 & 0 & 0 & 0 & 1 & 1 & 0 \end{array}\right]$$

The leader–follower connection is given by

(37) $$\beta =\left[\begin{array}{cccccccccc} 0 & 1 & 0 & 0 & 0 & 0 & 0 & 1 & 0 & 1 \end{array}\right]$$

β(i);i=1,2,…,N denotes the connection between leader with *i*th follower agent. β shows that the leader is connected to follower agents 2,8, and 10.

### 6.3. Results and Discussion: Fixed Topology

We have considered two cases to describe the controller’s performance. They are discussed in the following section.

#### 6.3.1. Case 1: Leader States-Constant and Ramp Function

In this case, the leader dynamics are given as follows.

(38) $$\begin{array}{ccc} {\dot{X}}_{L_{1}} & = & 1 \end{array}$$

(39) $$\begin{array}{ccc} {\dot{X}}_{L_{2}} & = & 0 \end{array}$$

**Figure 1:**
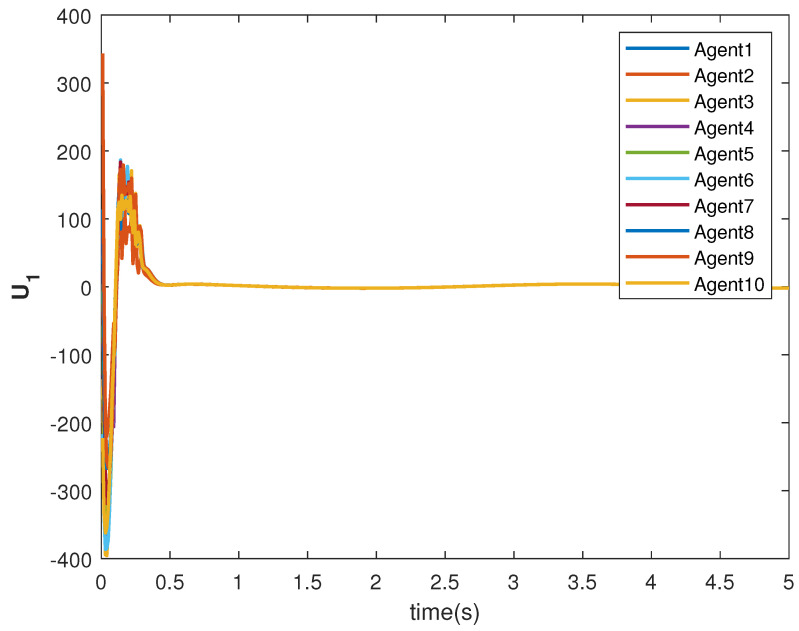
Control $U_{1}$ of agents.

**Figure 2:**
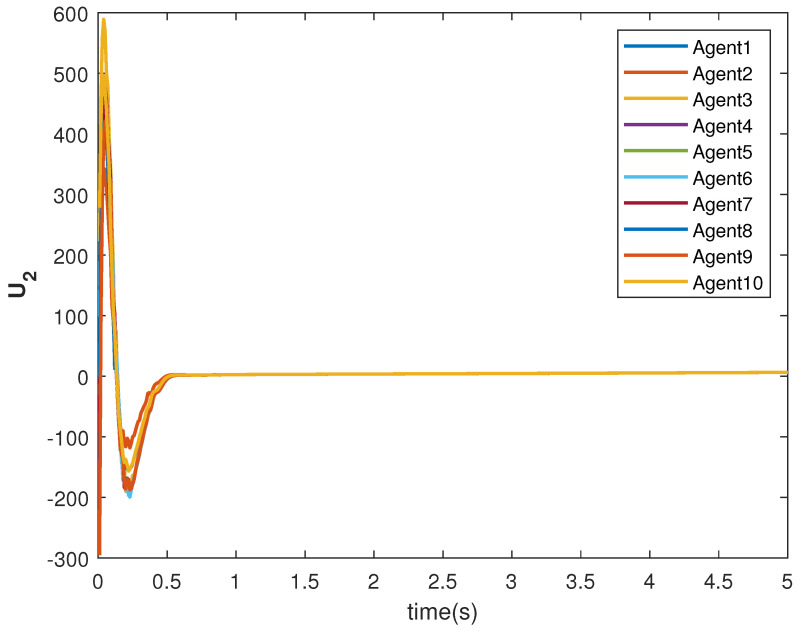
Control $U_{2}$ of agents.

**Figure 3:**
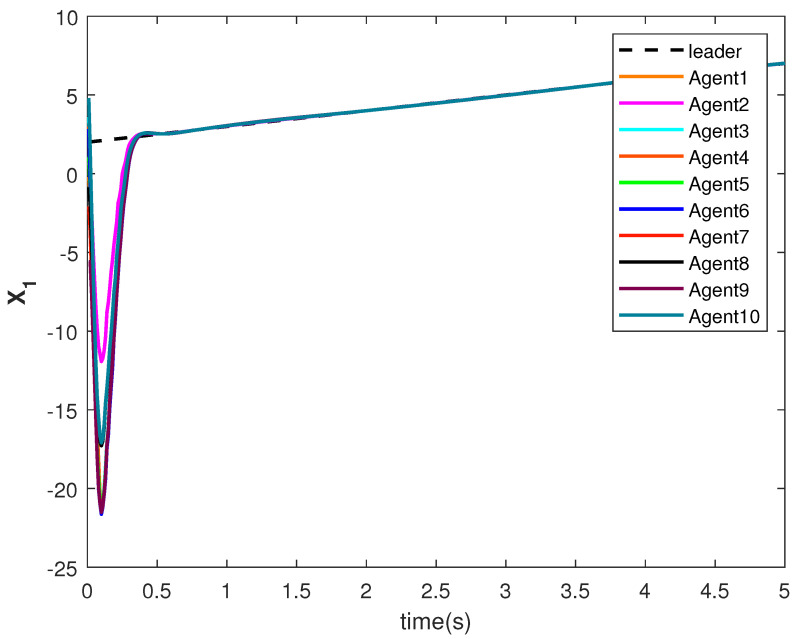
Consensus tracking of state $X_{1}$ of the agents.

**Figure 4:**
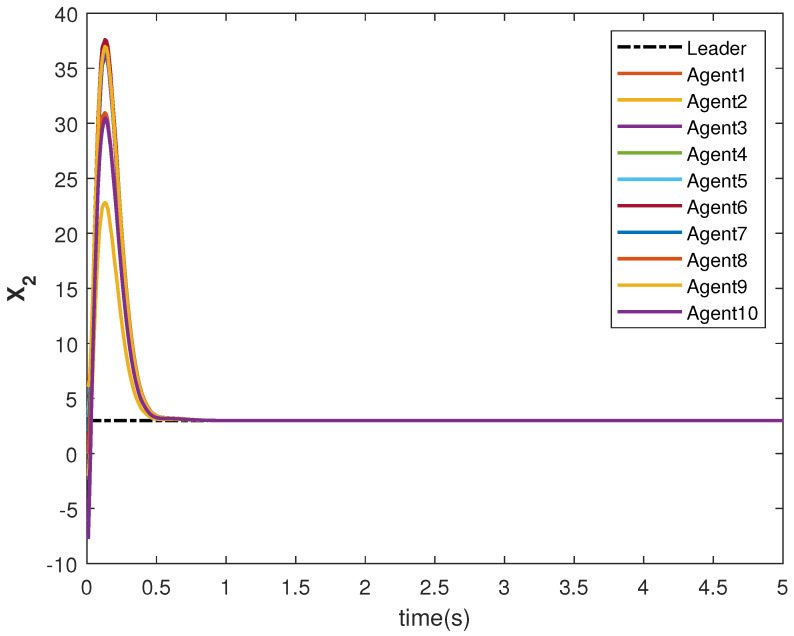
Consensus tracking of state $X_{2}$ of the agents.

**Figure 5:**
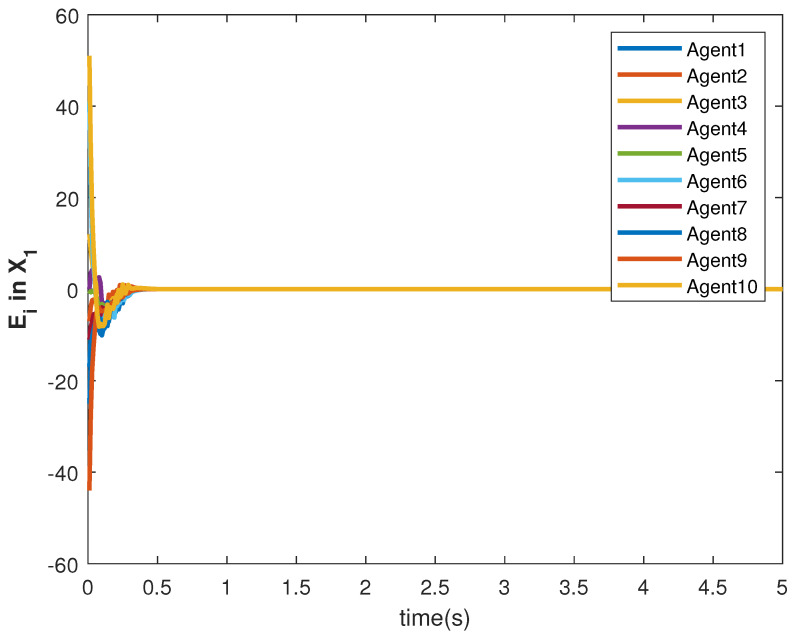
Consensus error $E_{i}$ in state $X_{1}$ of agents.

**Figure 6:**
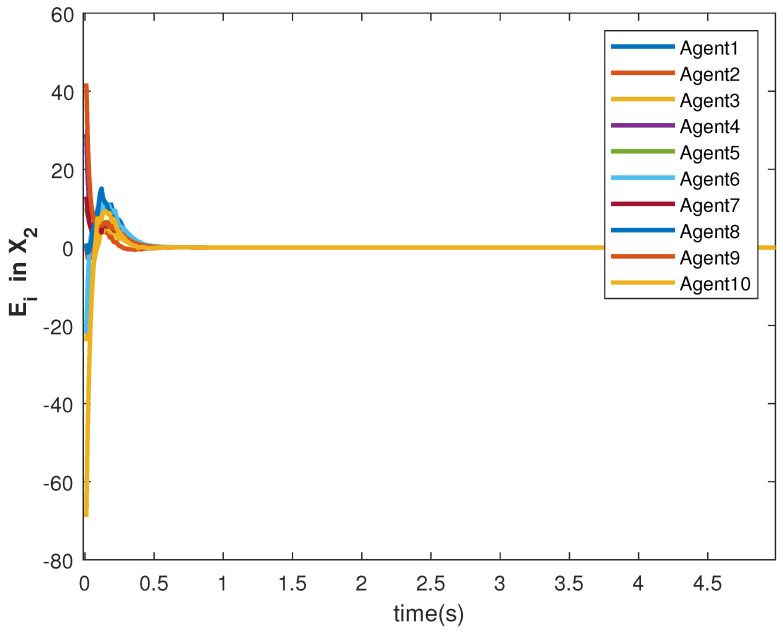
Consensus error $E_{2}$ in state $X_{1}$ of agents.

The consensus tracking controls $U_{1}$ and $U_{2}$, generated by the DNDI, are shown in Figure 1 and Figure 2, respectively. These controls produce the state trajectories. The states of the leader ($X_{L_{1}}$ and $X_{L_{2}}$) are ramp and constant functions, respectively. It can be seen that the agents’ states $X_{1}$ and $X_{2}$ track the leader states $X_{L_{1}}$ and $X_{L_{2}}$, respectively (shown in Figure 3 and Figure 4). The states achieve consensus with values dictated by the leader. The consensus error $E_{i}$ in states Figure 5 and Figure 6 shows the tracking accuracy.

#### 6.3.2. Case 2: Leader States-Sinusoid Function

In this case, the leader dynamics are considered as follows.

(40) $$\begin{array}{ccc} {\dot{X}}_{L_{1}} & = & 1.5X_{L_{1}}\left(t\right)\cos\left(2t+1\right)+X_{L_{2}}\left(t\right)\cos\left(4t\right) \end{array}$$

(41) $$\begin{array}{ccc} {\dot{X}}_{L_{2}} & = & 2X_{L_{2}}\left(t\right)\sin\left(3t\right) \end{array}$$

**Figure 7:**
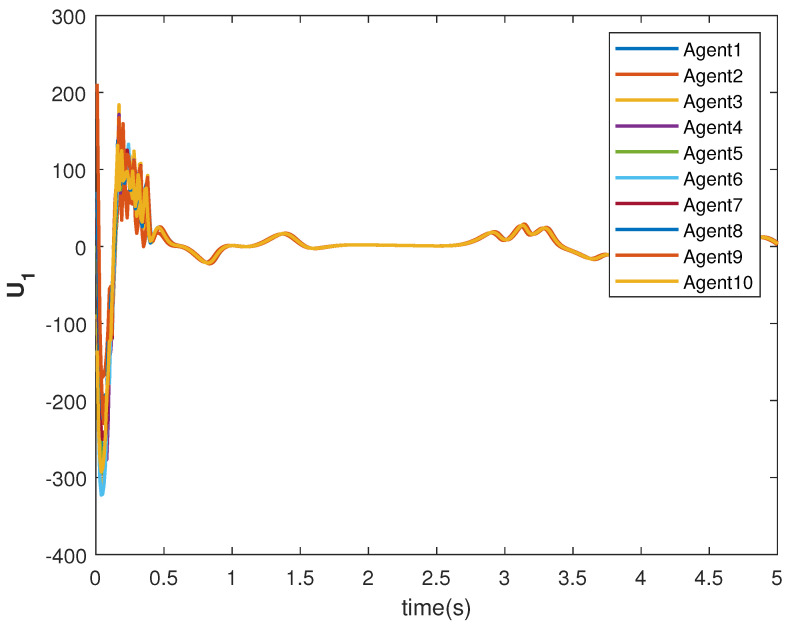
Control $U_{1}$ of agents.

**Figure 8:**
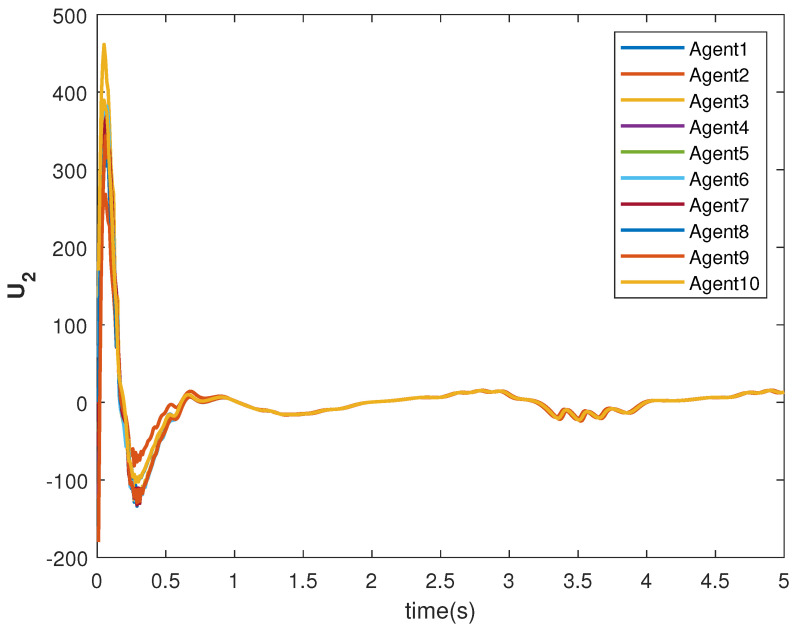
Control $U_{2}$ of agents.

The consensus controls $U_{1}$ and $U_{2}$ are shown in Figure 7 and Figure 8, respectively. It can be observed that the control signals are different from case 1. This is due to the leader’s states, which are sinusoid in nature as given in Equations (40) and (41).

**Figure 9:**
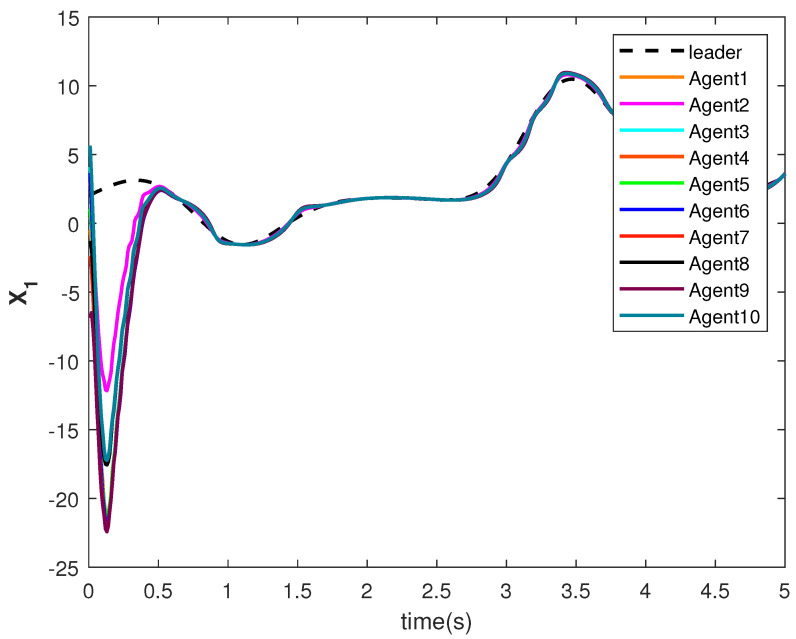
Consensus tracking of state $X_{1}$ of the agents.

**Figure 10:**
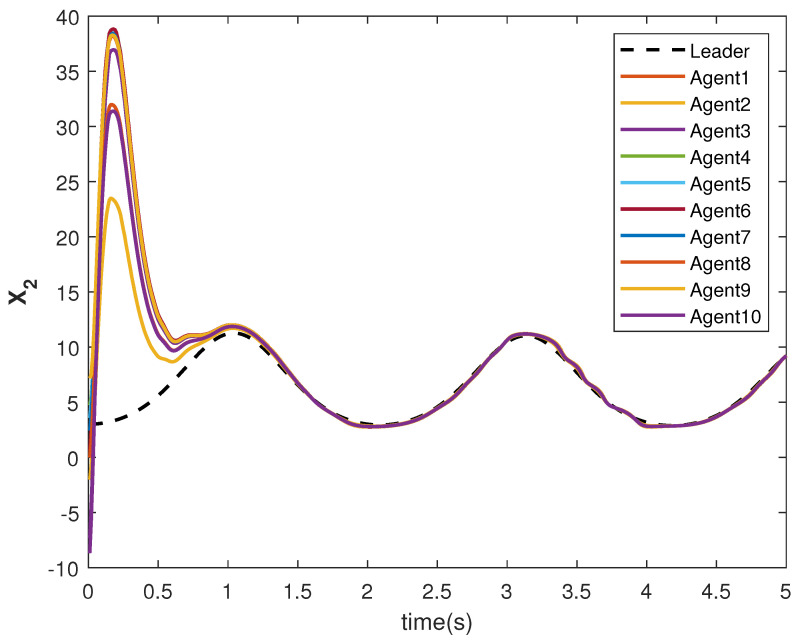
Consensus tracking of state $X_{2}$ of the agents.

The state trajectories $X_{1}$ and $X_{2}$ are shown in Figure 9 and Figure 10, respectively. The agents achieve consensus on the leader’s trajectories. The leader’s states are different, but the consensus controller has managed to track them.

**Figure 11:**
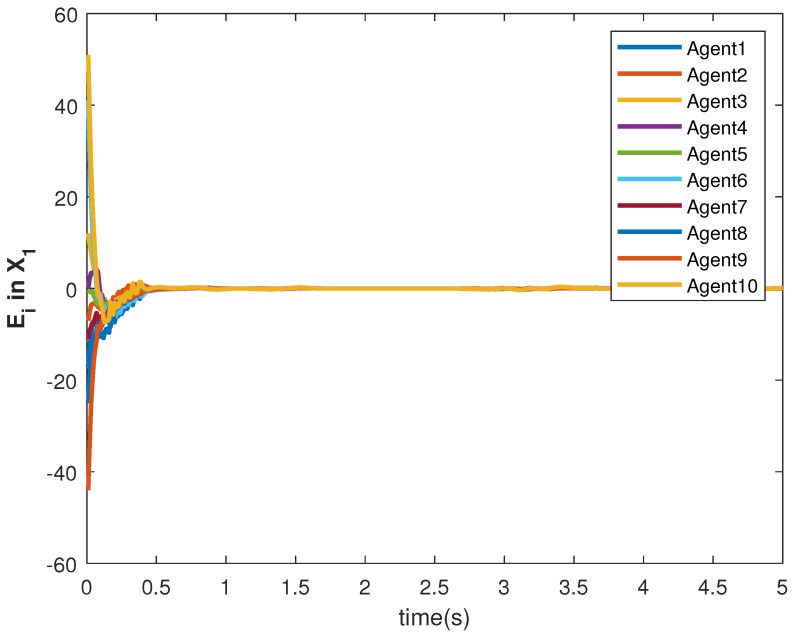
Consensus error $E_{i}$ in state $X_{1}$ of agents.

**Figure 12:**
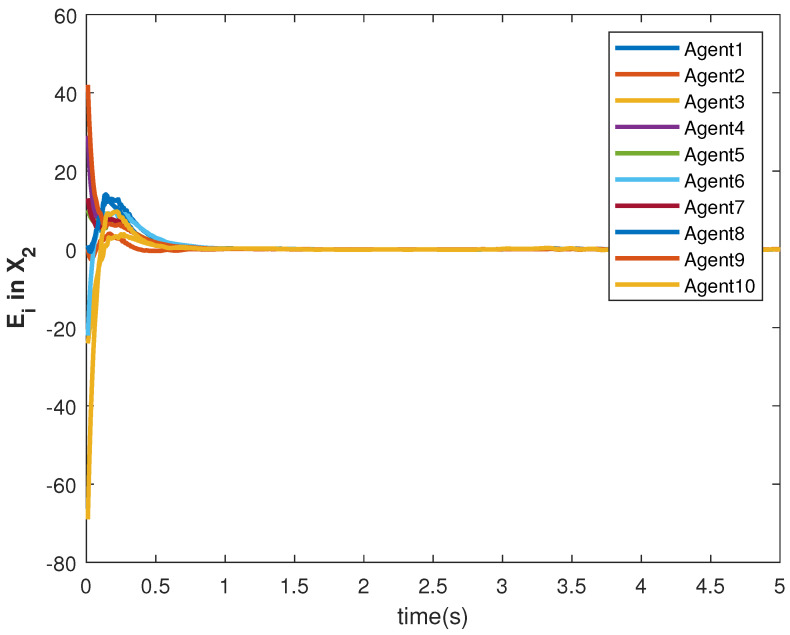
Consensus error $E_{2}$ in state $X_{1}$ of agents.

The accuracy of consensus tracking is described by the errors $E_{i}$ in $X_{1}$ and $X_{2}$, which are shown in Figure 11 and Figure 12, respectively. The consensus tracking errors become zero in a few seconds, which explains the effectiveness of the proposed controller.

### 6.4. Results and Discussion: Switching Topology and Switching Leader-Follower Connections

In this case, we have presented the case where both (a) switching topology among the agents and (b) switching connection between the leader and the followers. The switching topologies are generated by the Algorithm 1.
**Algorithm 1** Random topology generation.**for**k=1 to $N_{p}$ **do**   **for** i=1 to *N* **do**     **for** j=1 to *N* **do**        x← random number x∈(0,1)        **if** x>0.5 **then**          $A_{k}\left(i,j\right)\leftarrow 1$          $A_{k}\left(j,i\right)\leftarrow 1$        **else**          $A_{k}\left(i,j\right)\leftarrow 0$          $A_{k}\left(j,i\right)\leftarrow 0$        **end if**        **if** i=j **then**          $A_{k}\left(i,j\right)\leftarrow 0$        **end if**     **end for**   **end for****end for**

We have generated $N_{p}$ adjacency matrices, which denote the undirected topologies. *N* denotes the number of followers. The (i,j)th, i,j=1,2,…,N element of *k*th adjacency matrix $k=1,2,\ldots ,N_{p}$ is generated depending on the value of a random variable *x*, which is mentioned in the Algorithm 1. One topology at each time instant is selected (denoted by $A_{s}$) randomly (among $N_{p}$ topologies) using the Algorithm 2. A random integer ind in the range $\left[1,N_{p}\right]$ is selected, and the corresponding topology $A_{ind}$ is chosen as $A_{s}$.
**Algorithm 2** Selection of topology.**for**i=1 to $T_{s}$ **do**   x← random number x∈(0,1)   **if** x>0.5 **then**     $ind\leftarrow random\_integer(\left[1\;N_{p}\right],1)$     $A_{S}\leftarrow A_{ind}$   **else**     $A_{S}$ remains same   **end if****end for**

$T_{s}$ is the simulation time. Algorithms 1 and 2 were designed for implementing switching topology among the followers. Next, we will present the algorithms to describe the changing connections between the leader and the followers. $N_{L}$ leader–follower connections are generated using Algorithm 3. It can be observed that each element of the array temp is generated depending on the random variable *x* and a threshold value *l*. All the arrays generated are stored in the variable LF_con.
**Algorithm 3** Switching leader–follower connection.**for**i=1 to $N_{L}$ **do**   **for** j=1 to *N* **do**     x← random number x∈(0,1)     **if** x>l **then**        temp(j)←1     **else**        temp(j)←0     **end if**   **end for**   LF_con(i,:)←temp**end for**

**Figure 13:**
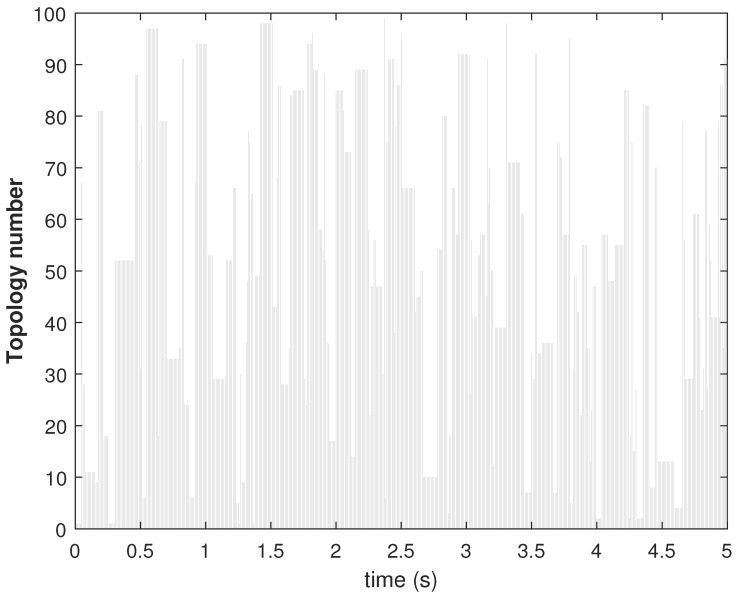
Consensus error $E_{i}$ in state $X_{1}$ of agents.

**Figure 14:**
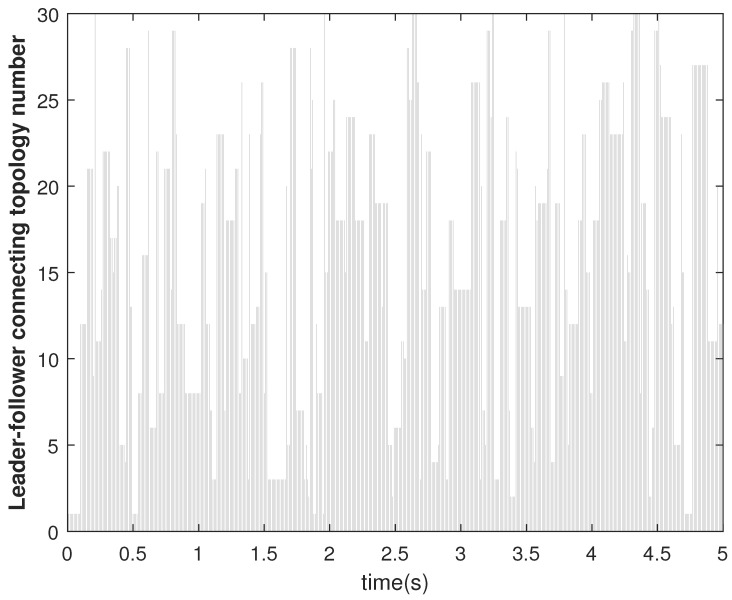
Consensus error $E_{2}$ in state $X_{1}$ of agents.

The leader–following switching connection is selected using the Algorithm 4. At each simulation time instant, one random integer ind is generated, and the array corresponding to ind in LF_con is selected as β. We considered the values of $N_{p}$ and $N_{L}$ as 100 and 30, respectively. The switching of topologies among the agents and switching connection of the leader–follower are shown in Figure 13 and Figure 14, respectively. The topologies among the follower (given by the topology number) agents change at every time instant according to Algorithm 2. Similarly, the connections between the leader and the followers (given by the connection number) change according to Algorithm 4.
**Algorithm 4** Selection of leader–follower connection.**for**i=1 to $T_{s}$ **do**   x← random number x∈(0,1)   **if** x>l **then**     $ind\leftarrow random\_integer(\left[1\;N_{L}\right],1)$     β←LF_con(ind,:)   **else**     β remains same   **end if****end for**

**Figure 15:**
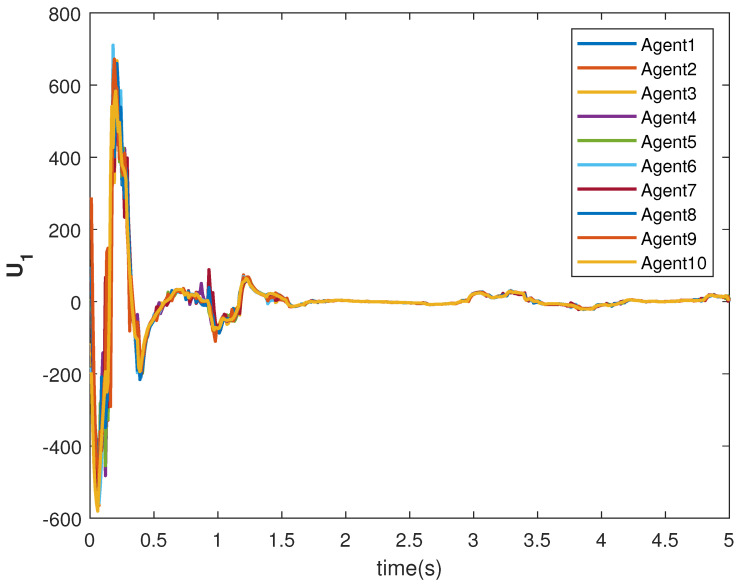
Control $U_{1}$ of agents.

**Figure 16:**
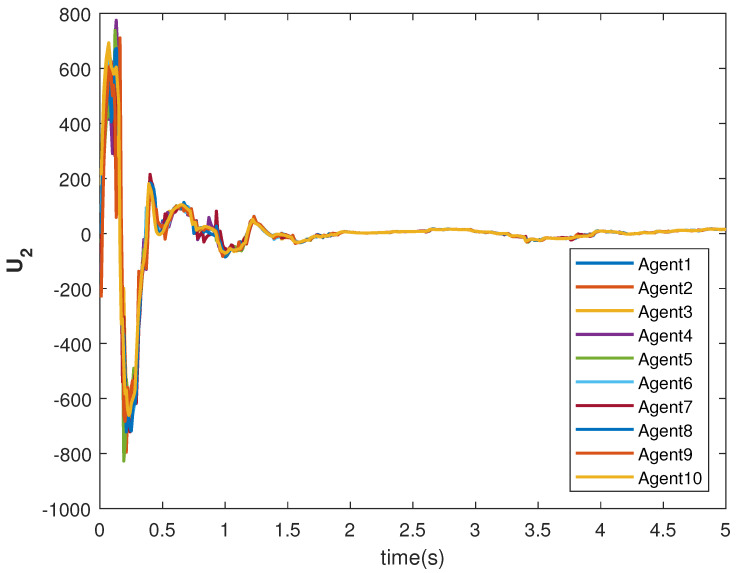
Control $U_{2}$ of agents.

The tracking consensus controls $U_{1}$ and $U_{2}$ are shown in Figure 15 and Figure 16, respectively. They have differences from other cases, which is the effect of the switching topology and connections.

**Figure 17:**
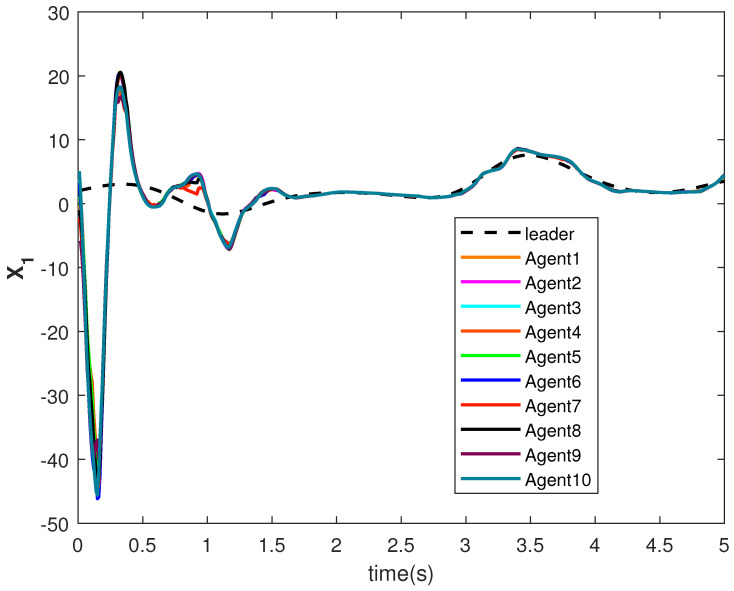
Consensus tracking of state $X_{1}$ of the agents.

**Figure 18:**
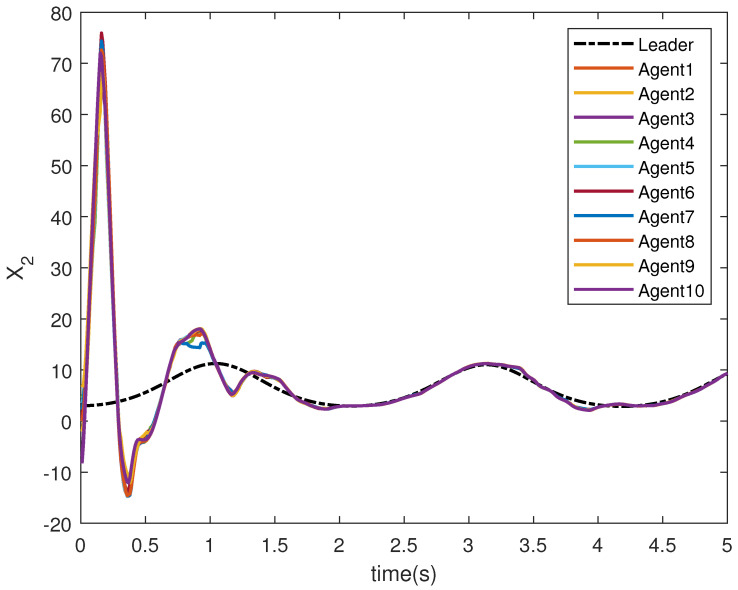
Consensus tracking of state $X_{2}$ of the agents.

**Figure 19:**
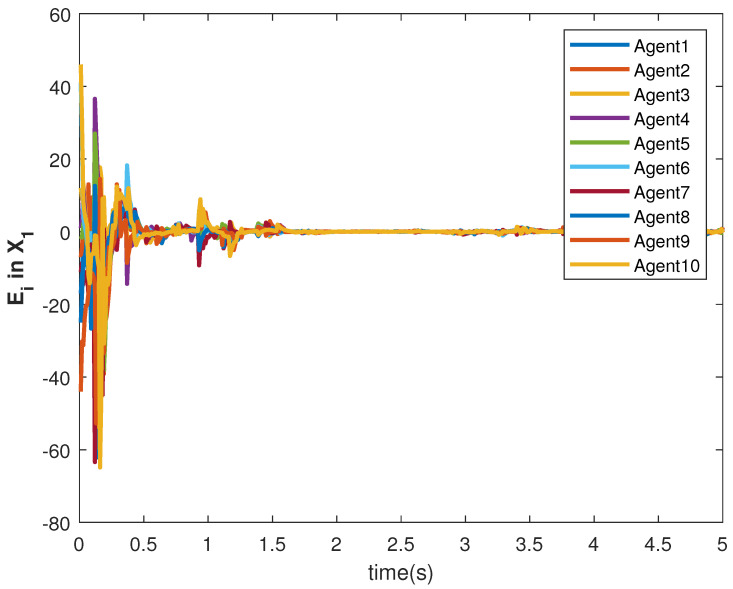
Consensus error $E_{i}$ in state $X_{1}$ of agents.

**Figure 20:**
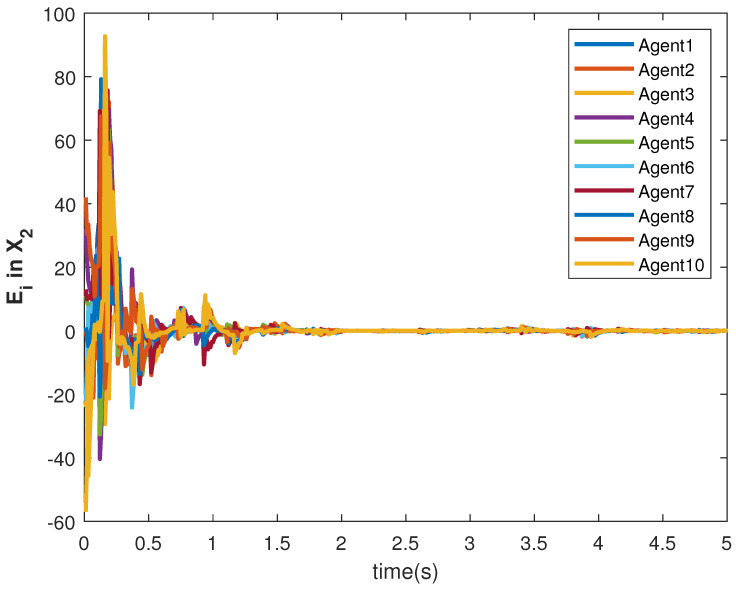
Consensus error $E_{2}$ in state $X_{1}$ of agents.

The state trajectories are generated by the control. The states of the followers started tracking efficiently within 2 s (see Figure 17 and Figure 18). The effect of the switching is more visible within this time. However, the DNDI-based controller managed to reduce the error (see Figure 19 and Figure 20) and improved the tracking performance. Therefore, it is clear that the proposed controller can perform the consensus tracking even in the presence of switching topology among followers and switching leader-follower connections.

## 7. Conclusions

The DNDI-based fault-tolerant controller has been used to solve the consensus tracking control of nonlinear agents for the first time. This derivation is different compared to our previous work about leaderless consensus control. Moreover, switching topology among the agents and switching leader–follower connections are considered, which is more realistic and addressed for the first time. A convergence study is presented to prove the tracking capability of the controller. A realistic simulation study evaluates the controller’s performance, where different types of leader trajectories are generated, and the agents successfully track the leader’s states. The results show that the proposed controller works efficiently in this realistic scenario. Therefore, the proposed controller is a potential candidate for consensus tracking applications.

## Data Availability

No new data were created or analyzed in this study. Data sharing is not applicable to this article.
